# Analysis of metatranscriptomic methods to enable wastewater-based biosurveillance of all infectious diseases

**DOI:** 10.3389/fpubh.2023.1145275

**Published:** 2023-03-22

**Authors:** Rachel R. Spurbeck, Lindsay A. Catlin, Chiranjit Mukherjee, Anthony K. Smith, Angela Minard-Smith

**Affiliations:** ^1^Health Business Unit, Drug Development and Precision Diagnostics Division, Life Sciences Research Business Line, Battelle Memorial Institute, Columbus, OH, United States; ^2^National Security Business Unit, Bioscience Center, CBRNE Business Line, Battelle Memorial Institute, Columbus, OH, United States; ^3^Health Business Unit, Health Analytics Division, Health Outcomes and Biotechnology Solutions Business Line, Battelle Memorial Institute, Columbus, OH, United States

**Keywords:** wastewater-based epidemiology, biosurveillance, infectious disease, metatranscriptomics, sequencing, genomics

## Abstract

**Introduction:**

Wastewater-based surveillance emerged during the COVID-19 pandemic as an efficient way to quickly screen large populations, monitor infectious disease transmission over time, and identify whether more virulent strains are becoming more prevalent in the region without burdening the health care system with individualized testing. Ohio was one of the first states to implement wastewater monitoring through its Ohio Coronavirus Wastewater Monitoring Network (OCWMN), originally tracking the prevalence of COVID-19 by quantitative qPCR from over 67 sites across the state. The OCWMN evolved along with the pandemic to include sequencing the SARS-CoV-2 genome to assess variants of concern circulating within the population. As the pandemic wanes, networks such as OCWMN can be expanded to monitor other infectious diseases and outbreaks of interest to the health department to reduce the burden of communicable diseases. However, most surveillance still utilizes qPCR based diagnostic tests for individual pathogens, which is hard to scale for surveillance of multiple pathogens.

**Methods:**

Here we have tested several genomic methods, both targeted and untargeted, for wastewater-based biosurveillance to find the most efficient procedure to detect and track trends in reportable infectious diseases and outbreaks of known pathogens as well as potentially novel pathogens or variants on the rise in our communities. RNA extracts from the OCWMN were provided weekly from 10 sites for 6 weeks. Total RNA was sequenced from the samples on the Illumina NextSeq and on the MinION to identify pathogens present. The MinION long read platform was also used to sequence SARS-CoV-2 with the goal of reducing the complexity of variant calling in mixed populations as occurs with short Illumina reads. Finally, a targeted hybridization approach was tested for compatibility with wastewater RNA samples.

**Results and discussion:**

The data analyzed here provides a baseline assessment that demonstrates that wastewater is a rich resource for infectious disease epidemiology and identifies technology gaps and potential solutions to enable this resource to be used by public health laboratories to monitor the infectious disease landscape of the regions they serve.

## 1. Introduction

Wastewater-based epidemiology (WBE) is a technique that extracts, analyses, and interprets targets excreted from feces or urine in wastewater to provide comprehensive community health information (1). Since wastewater is a population based sample, the whole community can be analyzed using one sample instead of necessitating individual sample collections, saving valuable time and resources. WBE was originally utilized for analysis of drug use in communities; the first reported application was the quantification of cocaine use in wastewater samples (2), and has been used world-wide since for illicit drug monitoring. Utilization of WBE for infectious disease surveillance began prior to the COVID-19 pandemic, however, the pandemic dramatically increased this use case. WBE has been utilized for pathogens such as enterovirus, arboviruses, polio, norovirus, *Salmonella*, enterohemorrhagic *Escherichia coli*, and giardia in wastewater and demonstrated the efficacy of WBE to identify outbreaks of these important communicable diseases (3–14). During the COVID-19 pandemic, WBE was implemented around the world to track viral spread throughout communities, demonstrating the power of testing community derived samples to follow disease transmission over time. As of March 11, 2021, the COVIDPoops19 global dashboard for wastewater monitoring of SARS-CoV-2 included 235 universities, 59 dashboards, and 1,488 sites in 55 countries, demonstrating the wide uptake of this method for tracking the COVID-19 pandemic (15). However, this implementation, like the utilization of WBE in other communicable disease surveillance efforts, is specific to surveillance of one pathogen at a time, detecting and quantifying the SARS-CoV-2 virus through quantitative polymerase chain reaction (qPCR) or digital droplet PCR (ddPCR). Identifying pathogens one at a time is reactionary and too slow to enable prevention of another pandemic. The COVID-19 pandemic has demonstrated the need for an active surveillance system to detect and identify infectious diseases before they spread across the world.

High throughput sequencing of pathogens within wastewater is one potential method that could enable surveillance of multiple pathogens simultaneously. However, most studies sequencing wastewater were again focused on the COVID-19 pandemic, utilizing targeted sequencing methods to identify sequence variants of the causative virus, SARS-CoV-2 (16–20). Furthermore, untargeted analyses tend to utilize DNA based metagenomics or 16S sequencing, removing any possibility of detection of RNA viral pathogens (21–23). In Ohio, wastewater tracking was established early on in the pandemic by the Ohio Department of Health in collaboration with several universities, government agencies, and non-profit research institutions as the Ohio Coronavirus Wastewater Monitoring Network (OCWMN, https://coronavirus.ohio.gov/dashboards/other-resources/wastewater). Ohio was one of the first states to implement wastewater monitoring, originally tracking prevalence of COVID-19 by quantitative qPCR from over 67 wastewater treatment facilities across the state. The OCWMN evolved along with the pandemic to include sequencing the SARS-CoV-2 genome to assess variants of concern circulating within the population. However, as the pandemic wanes, WBE networks like the OCWMN could transition its monitoring of SARS-CoV-2 to other communicable diseases monitored by the health department to aid infectious disease epidemiology and community health alerts. To determine the best method for tracking communicable diseases in wastewater, RNA samples from the OCWMN were provided over 6 weeks from ten different locations.

To unlock the power of WBE to provide a wholistic understanding of the communicable disease landscape within a region, the method to detect pathogens must be high throughput and multiplexed to detect any human pathogen. It is imperative to determine how to assess all bacterial, viral, or fungal human pathogens in wastewater samples simultaneously to prevent future outbreaks, whether at the city, state, national, or global scale. Previously, we demonstrated metatranscriptomic sequencing (analysis of total RNA from wastewater) was able to detect multiple pathogenic bacteria and viruses simultaneously (20, 24), however, this method is limited due to the need for ultradeep sequencing to detect viral pathogens. Therefore, we undertook this study to determine the most effective high throughput sequencing method for pathogen tracking in wastewater, comparing metatranscriptomic sequencing by Illumina, MinION, and also hybridization-based sequencing methods. As the pandemic wanes, the wastewater tracking networks set up worldwide will begin to shut down or will need to transition to surveillance of other pathogens to increase utility. Furthermore, sample preparation methods can influence the ability of sequencing to detect all pathogens, and therefore, we have included analysis of viral concentration and RNA extraction effects on sequencing results to help determine the most effective method for pan-pathogen biosurveillance. We have focused on RNA sequencing methods, as most WBE networks worldwide have been focused on SARS-CoV-2, an RNA virus. By utilizing RNA, methods identified in this work can be readily implemented within existing infrastructure to survey all human pathogens. Through this work, we endeavor to enable prevention of future outbreaks and pandemics through active surveillance in wastewater and identify gaps in laboratory procedures which need to be addressed for sequencing-based surveillance to be most effective.

## 2. Materials and methods

### 2.1. Samples

RNA extracts were sent to Battelle each week for 6 weeks from the OCWMN members who had conducted the quantitative PCR for SARS-CoV-2 viral load. The 10 sampling sites were chosen to span the five RNA extraction methods used as well as population sizes covered in the network. Table 1 lists the facility, site ID, RNA extraction method, and population served at each site assessed by Battelle.

**Table 1:** RNA extraction methods and populations (2012 census) for sample sites in the study.

**Site**	**RNA extraction method**	**Approximate population served (# people)**
A	1. membrane recombined with separated solids, TRIzol and RNA purification kit	365,000
B		54,000
C	2. centrifugation and membrane filtration, no additions, Qiagen PowerWater kit or Qiagen AllPrep kit	323,000
D	3. Qiagen QIAamp buffers with Epoch columns	11,000
E	4.5% Tween-20 amendment, concentrating pipette ultrafiltration, Qiagen PowerMicrobiome kit or Qiagen AllPrep PowerViral DNA/RNA	25,000
F		650, 000
G		45,000
H		655,000
I	5. Membrane filtration with no amendment, TRIzol, garnet bead beating, alcohol precipitation	46,000
J		65,000

### 2.2. RNA quality checks

Each collection week, RNA quantity and quality were measured using ThermoScientific NanoDrop 2000 Spectrophotometer (Cat# ND-2000) and Agilent 2100 Bioanalyzer (Cat# G2939BA) with an RNA 6000 Nano kit (Life Tech Cat# 5067-4627) following manufacturer's instructions. Samples exceeding 50 ng/μL on the NanoDrop were diluted before being analyzed on the Bioanalyzer to generate RNA integrity numbers (RIN, Table 2).

**Table 2:** RNA quantity and quality metrics upon receipt and after DNA removal.

**A**	**Receipt (ng/μL)**	**RIN**	**Post clean (ng/μL)**	**G**	**Receipt (ng/μL)**	**RIN**	**Post clean (ng/μL)**
**12/5/2021**	1,278.9	9.0	516.7	**12/5/2021**	6.8	0.0	3.6
**12/12/2021**	1,050.6	9.6	94.5	**12/12/2021**	18.6	0.0	ND
**12/19/2021**	972.8	7.5	231.8	**12/19/2021**	ND	2.1	1.7
**12/26/2021**	729.4	8.7	375.1	**12/26/2021**	4.1	1.0	4.7
**1/2/2022**	608.8	8.8	409.5	**1/2/2022**	16.6	1.5	13.0
**1/9/2022**	743.1	8.6	493.0	**1/9/2022**	6.5	1.0	5.3
**Mean**	897.3	8.7	353.4	**Mean**	10.5	0.9	5.7
**st dev**	249.0	0.7	162.2	**st dev**	6.6	0.8	4.3
**E**	**Receipt (ng/**μ**L)**	**RIN**	**Post clean (ng/**μ**L)**	**H**	**Receipt (ng/**μ**L)**	**RIN**	**Post clean (ng/**μ**L)**
**12/5/2021**	4.8	0.0	3.5	**12/5/2021**	5.3	1.0	4.8
**12/12/2021**	12.3	0.0	ND	**12/12/2021**	6.8	0.0	ND
**12/19/2021**	ND	1.0	2.5	**12/19/2021**	ND	0.0	1.9
**12/26/2021**	2.6	0.0	2.0	**12/26/2021**	2.4	0.0	2.2
**1/2/2022**	3.5	0.0	1.3	**1/2/2022**	3.4	0.0	5.1
**1/9/2022**	5.4	0.0	2.5	**1/9/2022**	5.1	0.0	3.6
**Mean**	5.7	0.2	2.4	**Mean**	4.6	0.2	3.5
**st dev**	3.8	0.4	0.8	**st dev**	1.7	0.4	1.5
**I**	**Receipt (ng/**μ**L)**	**RIN**	**Post clean (ng/**μ**L)**	**J**	**Receipt (ng/**μ**L)**	**RIN**	**Post clean (ng/**μ**L)**
**12/5/2021**	4,667.3	0.0	423.1	**12/5/2021**	3,872.5	0.0	107.9
**12/12/2021**	1,819.4	N/A	269.4	**12/12/2021**	2,812.0	N/A	246.5
**12/19/2021**	2,150.2	6.2	390.6	**12/19/2021**	2,097.2	6.0	324.3
**12/26/2021**	2,477.5	N/A	1,628.8	**12/26/2021**	1,602.3	N/A	457.4
**1/2/2022**	1,009.9	7.4	428.0	**1/2/2022**	1,302.0	2.9	690.3
**1/9/2022**	2,010.0	0.0	889.5	**1/9/2022**	1,120.1	6.0	426.9
**Mean**	2,355.7	3.4	671.6	**mean**	2,134.4	3.7	375.6
**st dev**	1,234.4	4.0	514.9	**st dev**	1,047.5	2.9	199.7
**F**	**Receipt (ng/**μ**L)**	**RIN**	**Post clean (ng/**μ**L)**	**B**	**Receipt (ng/**μ**L)**	**RIN**	**Post clean (ng/**μ**L)**
**12/5/2021**	4.3	0.0	3.5	**12/5/2021**	1,174.1	8.0	217.1
**12/12/2021**	12.5	1.0	ND	**12/12/2021**	425.9	8.9	63.4
**12/19/2021**	ND	1.2	2.4	**12/19/2021**	1,159.2	8.4	454.0
**12/26/2021**	2.4	0.0	10.4	**12/26/2021**	465.6	8.1	420.6
**1/2/2022**	2.9	0.0	6.5	**1/2/2022**	89.9	7.9	34.8
**1/9/2022**	6.8	0.0	7.2	**1/9/2022**	878.3	8.1	738.6
**mean**	5.8	0.4	6.0	**mean**	698.8	8.2	321.4
**st dev**	4.1	0.6	3.2	**st dev**	440.4	0.4	268.7
**D**	**Receipt (ng/**μ**L)**	**RIN**	**Post clean (ng/**μ**L)**	**C**	**Receipt (ng/**μ**L)**	**RIN**	**Post clean (ng/**μ**L)**
**12/5/2021**	57.7	0.0	16.2	**12/5/2021**	513.8	3.4	222.9
**12/12/2021**	96.6	2.3	44.4	**12/12/2021**	79.2	2.8	25.5
**12/19/2021**	84.8	2.8	118.9	**12/19/2021**	93.2	5.8	33.2
**1/9/2022**	88.5	4.7	53.8	**1/2/2022**	155.6	2.5	102.3
**Mean**	81.9	2.5	58.3	**1/9/2022**	353.6	3.3	274.0
**st dev**	16.9	1.9	43.4	**Mean**	239.1	3.6	131.6
				**st dev**	188.7	1.3	112.3

ND, not detected; N/A, not available data.

### 2.3. Subtractive hybridization and DNase treatment

Some samples provided by the OCWMN contained a mixture of RNA and DNA, enabling the reduction of unwanted, high abundance nucleic acids through subtractive hybridization. This method utilizes hybridization between highly abundant RNAs and their DNA counterpart in the same sample, followed by RNaseH treatment to degrade the abundant RNAs and a DNase step to remove the DNA prior to sequencing. The objective of utilizing subtractive hybridization in this case was to remove human RNAs and highly abundant non-pathogenic bacterial RNAs to enrich for pathogens such as viruses that are less abundant in the samples. Briefly, samples from weeks 1 and 2 were RNase treated by combining 5 μL of sample with a mixture of 0.5 μL of RNase H and 5 μL of RNaseH Buffer and incubated at 37°C for 20 min. A Zymo RNA clean and concentrate kit (Cat# R1013) was used to increase RNA yield. For weeks 3–6, which did not have subtractive hybridization performed, an optional DNase step was included in the Zymo RNA clean and concentrate procedure following the manufacturer's instructions. Following this procedure, a second nanodrop was performed to assess and compare RNA quantities from receipt. The concentration of nucleic acid before and after concentration and the RIN score upon receipt is shown in Table 2 for all samples used in the study.

### 2.4. Untargeted RNA sequencing on Illumina NextSeq

RNA was treated using the Swift Rapid RNA Library Kit (Cat# R2096) according to the manufacturer's instructions with the following modifications. After the indexing amplification, libraries were quality checked for size distribution using the Agilent 2100 Bioanalyzer and an Agilent High Sensitivity DNA Kit (Agilent Cat#5067-4626). For many samples, a sharp adapted primer peak of ~130 bp was observed, at a much higher concentration than the actual library molecules. Sequencing of libraries without removal of this peak caused the sequencing run to fail. Therefore, size selection methods were utilized to remove the peak prior to sequencing when the ~130 bp peak was >150–200 FU. Two size selection methods were used. For weeks 1 and 2, size selection was conducted by two SPRISelect bead cleanups at 0.8X bead to library volume. This method was very time consuming and not precise enough to remove all the adapted primer peak. Therefore, starting on week 3, samples were processed using the Blue Pippin (Sage Science Cat# BLU0001) to perform size selection. This method was automated and more precise than the bead clean up, therefore was used for all samples moving forward. Using 2% gel cassette kits (Sage Science Cat# BEF2010), samples were automatically size selected to between 200 and 600 BP. Removal of the unwanted adapted primer peak was confirmed by the Agilent High Sensitivity DNA kit prior to library quantification by KAPA Library Quant Kit (Illumina) Universal qPCR Mix (Cat# KK4824) following the manufacturer's instructions. Libraries were normalized to 4 nM and pooled for sequencing on the Illumina NextSeq 500/550 on High Output Kits v2.5 (300 Cycles, 150 × 150 bp).

Data was analyzed using two bioinformatic approaches to identify pathogens present: a k-mer based approach and a contig based approach. In the k-mer based approach, demultiplexed paired FASTQ files pertaining to each sample were processed using a custom short-read classification pipeline. Briefly, raw reads were adapter trimmed and quality filtered using Trimmomatic (v0.39) (25). The K-mer based classification tool Kraken2 (v2.1.2) (26) was used to assign taxonomy to the remaining host-filtered reads, using a reference database containing NCBI's RefSeq genomes covering bacterial, archaeal, viral, fungal, human and protist taxonomy (Kraken2 database obtained from https://benlangmead.github.io/aws-indexes/k2, version “Plus PF” created 05/17/21). Downstream data analysis and visualizations were performed using the statistical software R. Two methods were used to minimize false positives arising due to use of short reads and a broad database:

1. Kraken2 confidence score cutoff of 0.25 was used to set the threshold for number of k-mers mapping to LCA value in the clade rooted at that label.
2. A 0.1% relative abundance cutoff was used to discard ultra-low abundance species matches likely arising due to limitations of short read based taxonomic classification.

In the contig based approach to analyze the metatranscriptomic data, the RNA-Seq files were demultiplexed, quality checked, and trimmed. Sequences were assembled using SPAdes (27) into contigs and then compared to known reference sequences in the refseq database using BlastN or DIAMOND BlastX (28) to identify pathogens in the wastewater samples. Similar to the K-mer based approach, a 0.1% relative abundance cutoff was used to discard ultra-low abundance species hits before determining the organism present in the dataset.

### 2.5. Untargeted RNA sequencing on MinION using direct RNA sequencing Kit

Six samples were prepared for Direct RNA Sequencing on the Oxford Nanopore Technologies MinION portable sequencer following the manufacturer's instructions. Two samples A 1/09/22 and H 12/26/21 were sequenced on the MinION and data analyzed in real time using the ONT What's in My Pot? Application (WIMP). Three WIMP options were used to analyze both samples: Fastq WIMP, Fastq Antimicrobial Resistance WIMP, and Fastq WIMP (Human + Viral). The minimum abundance output for the NCBI Taxonomy Tree was set at 1% for all analyses.

### 2.6. Midnight RT PCR expansion pack for MinION sequencing of SARS-CoV-2

The whole genome of SARS-CoV-2 was sequenced from all 55 samples using the Midnight RT PCR Expansion Pack for MinION. This kit was chosen, as the amplicons are longer than those in the amplicon sequencing kits for Illumina, to see if longer amplicon sequences would enable better resolution of variants within the wastewater samples. Data was analyzed in real time using the MinION application MinKNOW and EPI2ME.

### 2.7. Illumina respiratory pathogen and AMR targeted sequencing panel

Twenty-eight samples and a SARS-CoV-2 positive control sample were prepared and sequenced following the manufacturer's instructions using the Illumina Respiratory Pathogen and AMR targeted hybridization sequencing kit. Data was demultiplexed and then transferred to BaseSpace where it was analyzed using the IDbyDNA Explify application.

### 2.8. Statistical analysis

Statistical analyses such as Student's *T*-tests were conducted in Excel to determine the significance of effects by population size and extraction methods on RNA quality and concentration.

## 3. Results

### 3.1. Extraction method influence on nucleic acid concentration and RNA quality

**Figure 1:**
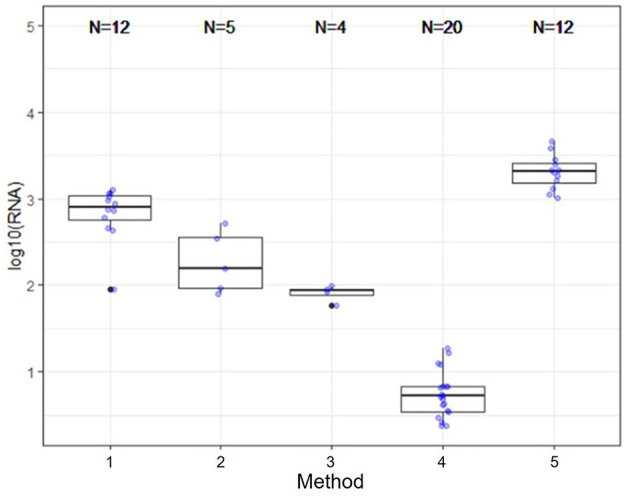
Extraction and viral concentration method significantly effects RNA concentration. While all five methods are capable of detecting SARS-CoV-2 by qPCR, as demonstrated by the data reported by the OCWMN, only methods 2 and 3 produced similar RNA concentrations. Therefore, metatranscriptome results could be highly variable between sites due to differences in extraction method.

**Figure 2:**
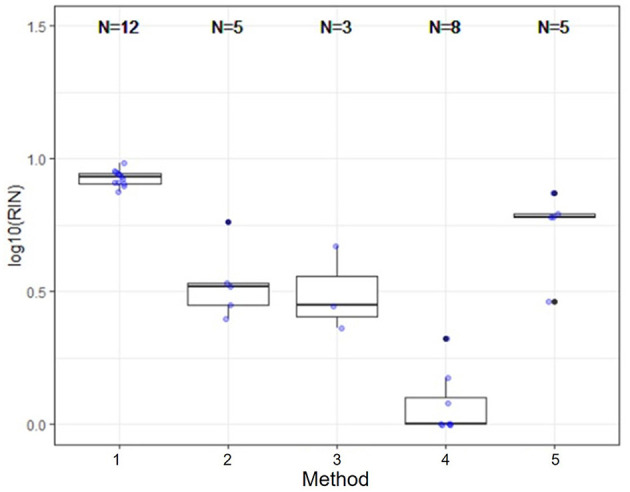
Extraction method significantly effects RNA integrity. RNA integrity was significantly different for all comparisons except between methods 2, 3, and 5. Therefore, extraction method may significantly impact the outcome of RNA sequencing.

Several nucleic acid extraction methods were utilized by the network due to supply chain issues during the pandemic. To enable comparison of data over time, the methods utilized at the beginning of the pandemic by each sewershed were continued throughout the monitoring period. Therefore, comparisons between sewersheds must be conducted with the understanding of the extraction methods utilized. Of the 10 locations analyzed on this study, five different extraction methods were used (Table 1): 1. Filtered membrane recombined with separated solids followed by TRIzol RNA purification 2. Wastewater was centrifuged to remove solids, membrane filtered, and the Qiagen PowerWater kit or Qiagen AllPrep kit used on the filtrate 3. Qiagen QIAamp buffers with Epoch columns were used on wastewater 4. 5% Tween-20 amendment, concentrating pipette ultrafiltration followed by Qiagen PowerMicrobiome kit or Qiagen AllPrep PowerViral DNA/RNA 5. membrane filtration with no amendment, TRIzol with garnet bead beating for RNA extraction, followed by alcohol precipitation to concentrate RNA. The extraction method utilized has a significant effect on the nucleic acid concentration as shown in Figure 1 and Table 3, with only methods 2 and 3 producing similar concentrations. Method 4 produces significantly less material than the other methods as shown in Table 3, due to the concentration of viral particles by the concentrating pipette ultrafiltration. Similarly, extraction method also influences the quality of the RNA extracted when comparing RIN scores as shown in Figure 2 and Table 3. RNA integrity was significantly different for all comparisons except between methods 2, 3, and 5. Therefore, extraction method may significantly impact the outcome of RNA sequencing.

**Table 3:** Extraction method influences RNA integrity number (RIN) score.

**Extraction method comparison**	**Nucleic acid concentration**			**RIN score**		
	**Test statistic**	**Confidence interval**	**Adjusted** ***P*****-value**	**Test statistic**	**Confidence interval**	**Adjusted** ***P*****-value**
1 vs. 2	3.7	1.4, 9.7	0.0023	2.5	1.6, 3.7	< 0.0001^*^
1 vs. 3	8.4	3.0, 23.9	< 0.0001^*^	2.7	1.6, 4.5	< 0.0001^*^
1 vs. 4	123.2	63.6, 238.8	< 0.0001^*^	7.2	5.0, 10.2	< 0.0001^*^
1 vs. 5	0.3	0.2, 0.7	0.0007^*^	1.6	1.0, 2.3	0.0294^*^
2 vs. 3	2.3	0.7, 7.7	0.5132	1.1	0.6, 1.9	1.0
2 vs. 4	33.4	13.5, 82.7	< 0.0001^*^	2.9	1.9, 4.5	< 0.0001^*^
2 vs. 5	0.1	0.0, 0.2	< 0.0001^*^	0.6	0.4, 1.0	0.0655
3 vs. 4	14.6	5.4, 39.5	< 0.0001^*^	2.6	1.6, 4.4	< 0.0001^*^
3 vs. 5	0.0	0.0, 0.1	< 0.0001^*^	0.6	0.3, 1.0	0.0537
4 vs. 5	0.0	0.0, 0.0	< 0.0001^*^	0.2	0.1, 0.3	< 0.0001^*^

Adjusted *P*-values (log by method). ^*^Indicates statistically significant values below 0.001.

### 3.2. Population size influence on nucleic acid concentration

RNA extraction methods 1 and 4 had data from both large populations and small populations which enabled analysis of the influence of population size on nucleic acid concentration within extraction method (Table 1). Method 1 was used on A (population 363,897, average RNA concentration 897.3 ± 249.0 ng/μL) and B (population 54,037, average RNA concentration 698.8 ± 440.4 ng/μL). Method 4 was used in four facilities covering two large populations: F (population 645,940) and H (population 654,817) with an average RNA concentration of 5.2 ± 3.0 ng/μL, and two small populations: E (population 24,536) and G (population 45,000) with an average RNA concentration of 8.1 ± 5.7 ng/μL. Within each method, the population size had no statistically significant effect on the RNA concentration (method 1: T-statistic 0.96, *P* = 0.36 and method 4: T-statistic −1.44, *P* = 0.17). This analysis suggests that population size covered by the sewershed samples does not influence the amount of nucleic acid concentration.

### 3.3. Midnight RT PCR expansion pack for MinION sequencing of SARS-CoV-2

One issue with genome sequencing of SARS-CoV-2 for variant of concern tracking in wastewater is the small amplicon size (~180 bp) of the Illumina based methods can confound viral variant calling, as the population sample can contain multiple viral variants simultaneously. Linkages between the defining mutations needed to differentiate between closely related viral variants may not be contained within the short amplicons. To determine if longer read sequencing on the MinION could help alleviate this issue, the genome of SARS-CoV-2 was sequenced from all samples using the Midnight RT-PCR Expansion Pack to enable longer read sequences (~1,200 bp) to be produced. Prior to sequencing samples, the amplicon product was quality checked on the Agilent Bioanalyzer. Samples which had a good amplification peak at 1,200 bp, were able to produce SARS-CoV-2 variant calls in real time on the MinION (Table 4). However, samples which did not have a visible 1,200 bp peak were unable to provide SARS-CoV-2 variant calls. Since this method relies on long, intact sequences, the RNA extraction method utilized impacts the ability to produce the long-read sequencing data. The four sites which consistently produced the 1,200 bp peak and SARS-CoV-2 variant calls on the MinION were all extracted by the same method (method 4): 5% Tween-20 amendment, concentrating pipette ultrafiltration, Qiagen PowerMicrobiome kit or Qiagen AllPrep PowerViral DNA/RNA kit and had the lowest concentrations of RNA compared to the other extraction methods. This suggests that these RNA extracts may have been more intact and enriched for viral sequences than those from other sites. Further work will need to be conducted using the different extraction methods on the same sample to determine the optimal extraction for long read sequencing.

**Table 4:** MinION SARS-CoV variant calls by date and site.

**Site**	**Consistent 1,200 bp peak (Y/N)**	**12/5/2021**	**12/12/2021**	**12/19/2021**	**12/26/2021**	**1/2/2022**	**1/9/2022**
A	N	NC	19A	NC	Delta (21J)	NC	NC
B	N	NC	NC	NC	NC	20B	NC
C	N	NC	NC	NC	NC	NC	NC
D	N	NC	NC	NC	NC	NC	Omicron (21M)
E	Y	Delta (21J)	Delta (21J)	Delta (21J)	20A	Omicron (21K)	Omicron (21K)
F	Y	Delta (21J)	Delta (21J)	Omicron (21M)	Omicron (21K)	Omicron (21K)	Omicron (21K)
G	Y	Delta (21J)	Delta (21J)	Delta (21J)	Omicron (21K)	Omicron (21K)	Omicron (21K)
H	Y	Delta (21J)	Delta (21J)	Delta (21J)	Omicron (21K)	Omicron (21K)	Omicron (21K)
I	N	NC	Delta (21J)	NC	NC	NC	NC
J	N	Delta (21J)	Delta (21J)	NC	NC	NC	NC
Positive	Y	19A	19A	19A			
Negative	N	NC	NC	NC			

NC, no call. Blue color highlights samples that consistently generated viral variant calls.

### 3.4. Untargeted RNA sequencing on illumina NextSeq

For each RNA sample, libraries were prepared using the Swift Rapid RNA Library kit and sequenced on the Illumina NextSeq in batches of 10 samples per run. It was hypothesized that subtractive hybridization, where DNA present in the RNA sample would bind to RNA, enabling RNaseH degradation of unwanted, highly prevalent nucleic acids, would enable enrichment of pathogenic viruses by reducing the background. Therefore, the first 2 weeks of samples were treated by subtractive hybridization using the DNA present in the RNA samples and analyzed to determine if human RNA viruses were better detected than in our previous work. However, no enrichment for viruses was observed by subtractive hybridization, suggesting that the DNA of highly prevalent human RNAs or non-pathogenic microbes was not present at high enough concentrations for effective removal. Therefore, subtractive hybridization was not used for weeks 3–6. Two bioinformatic methods were used to assess the data derived from the 55 samples: a K-mer based approach and a contig based approach. Both methods identified several pathogens in the samples, with some concerning hits to *Burkholderia pseudomallei* and *B. mallei*, biothreat agents not endemic to the Midwest region of the United States. Upon further analysis and literature review, it was determined that the genus *Burkholderia* contains several species that are difficult to differentiate through random sequence analysis, such as metatranscriptomics used on this project, and therefore, these hits were likely universal genomic regions common to other species of *Burkholderia* (29, 30).

#### 3.4.1. K-mer based analysis results

The k-mer approach identifies and quantifies species based on sequence identity to short fragments of nucleic acid sequences. Several questions were addressed using the k-mer based approach including:

1. Does RNA extraction method effect the microbial profile?
2. Does the RNA extraction method effect the amount of human RNA present in the samples?
3. What organisms from the Ohio Department of Health reportable infectious diseases list are detected in the samples?

**Figure 3:**
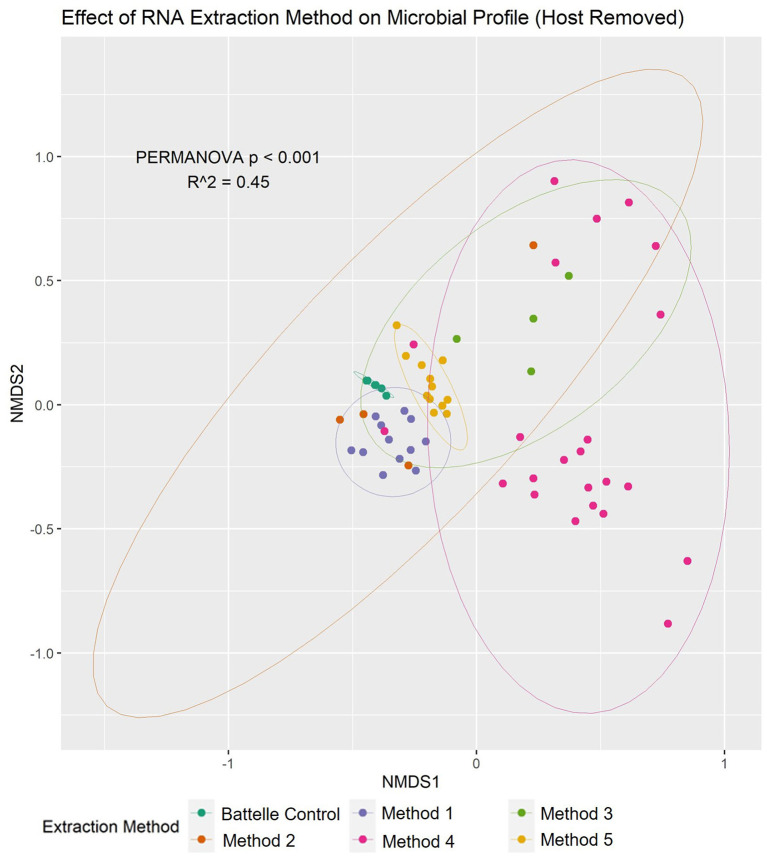
Nucleic acid extraction method had a significant effect on the wastewater microbiome profile based on NMDS and multivariate statistics. Non-metric multidimensional ordination plot showed that there was significant (PERMANOVA *p* < 0.001) difference between the microbiota composition of samples based on the method used to extract the RNA (shown in different colors). 95% confidence intervals for each method are also shown in the plot.

To determine the effect of extraction method on the microbial profile, non-metric multidimensional ordination and associated multivariate analysis (PERMANOVA) were conducted (Figure 3). There were significant differences in microbial profiles, with the extraction method being identified as a major contributor (R^2^ = 0.45) to this variation. Furthermore, when the human sequences present in each sample were quantified, method 4 was shown to have much higher proportions of the data being attributed to human (average 6.9 ± 15.8% range: 0.5–75.2%) compared to the other methods (average 0.5 ± 0.3%, range: 0.2–1.9%), suggesting that this method does not efficiently remove human cells prior to RNA extraction compared to the other methods.

**Figure 4:**
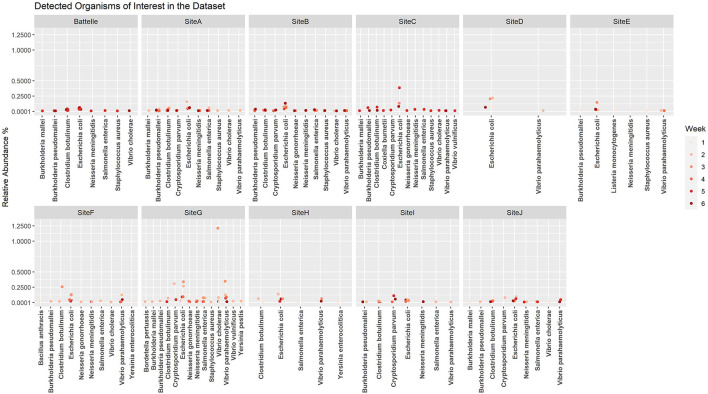
Relative abundance by site per week for etiological agents of Ohio Department of Health reportable diseases using the k-mer approach. A cutoff of 0.1% was used as a threshold for detection.

When the microbial fraction of each sample was analyzed, several etiological agents from the reportable infectious diseases list provided by ODH were identified and the proportion over time was able to be tracked at each location (Figure 4). To try to increase confidence in the data calls and reduce the number of ambiguous sequences such as those that map to *Burkholderia pseudomallei* or *B. mallei*, a cut off of 0.1% relative abundance was implemented. This reduced the total number of pathogens identified from each sample, as most pathogens were a minor fraction of the total sequence set. As seen from the scatter plots, most of these pathogens were only detected in ultra-low relative abundances, and only one organism in one sample was identified at a relative abundance over 1%. This was identified as *Vibrio cholerae* in G week 3. As this organism is generally a pathogen found in more tropical regions near the ocean, literature was again reviewed to determine if there was precedence for *V. cholerae* in Ohio. Indeed, *V. cholerae* isolates have been found in freshwater sources in northwest Ohio (31). While these strains are not from the serogroups (O1 and O139) that cause epidemic cholera, the isolates characterized in Daboul et al. contained a variety of virulence genes that could cause gastroenteritis or other human infections. Therefore, the *V. cholerae* hits in our wastewater analyses likely are real and could be studied to determine if there are trends of these organisms in regions with gastroenteritis or other enteric illness outbreaks. *Escherichia coli*, a microbe that can be found naturally in human and animal gut microbiomes, was one of the most commonly detected potential pathogens based on the k-mer data analysis. While *E. coli* can cause disease, the k-mer analysis cannot differentiate if the *E. coli* detected is pathogenic or commensal. A targeted approach that captures unique sequences from pathogens would be necessary to determine *E. coli* pathogenicity.

#### 3.4.2. Contig based analysis results

The second approach used to analyze the untargeted RNA sequencing data, the contig based analysis, pieces together as much of each organism's genome as possible prior to comparison to the sequence database for identification. Therefore, more sequence information is used, providing more accurate detection calls. However, this approach still does not avoid the issues of random sequence capture observed especially in the case of the genus *Burkholderia*, where many of the species contain similar genomic regions, and therefore endemic, non-pathogenic *Burkholderia* may be misidentified as *B. mallei* and *B. pseudomallei* in the wastewater samples.

Using the contig based approach, the proportions of the data identified as eukaryote, bacteria, archaea, or virus was assessed for each sample after the removal of human sequences. In all samples, most data aligned to bacteria. However, the samples from sites E (average 6.8 ± 2.9%), F (average 9.4 ± 2.1%), G (average 8.1 ± 7.9%), and H (average 8.2 ± 5.3%) (all extracted by method 4) demonstrated enrichment for viral sequences, reflective of the pre-processing these samples underwent prior to RNA extraction. Sites A, B, C, D, I, and J were not enriched for viral sequences (average 0.40 ± 0.42%). This difference was statistically significant by one-way ANOVA (F-statistic 9.2941, *P* = 8.46 x 10^−8^) and Tukey's HSD (honestly significant difference) posttest (*P* < 0.05 for each pair-wise comparison between sites extracted by method 4 and all other methods). However, this enrichment was not enough to enable detection of SARS-CoV-2 or other viral pathogens of public health concern from the untargeted sequencing data, and most viral sequences aligned to bacteriophage.

**Figure 5:**
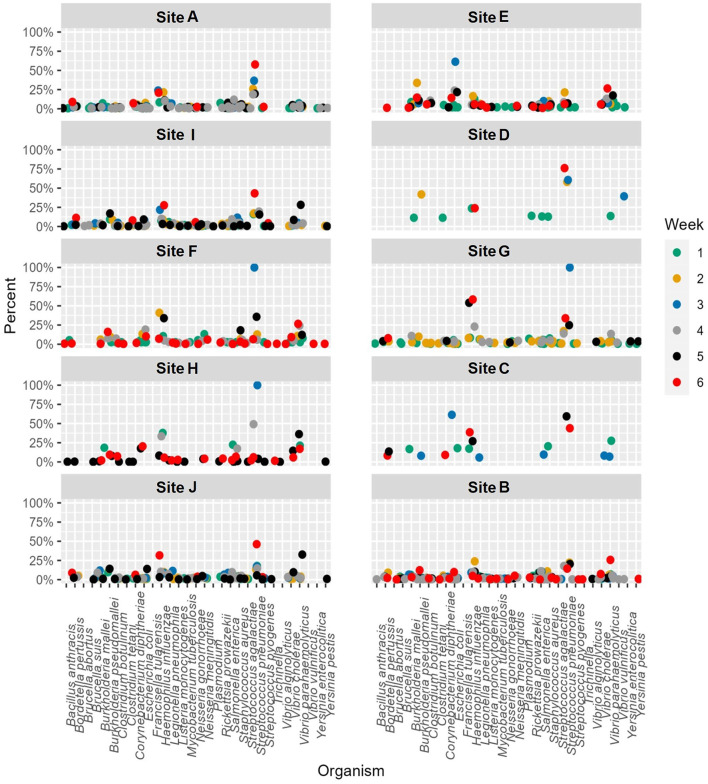
Abundance by site per week for etiological agents of Ohio Department of Health reportable diseases using the Contig-based approach to data analysis.

When the contig based approach was used to identify the etiological agents of ODH reportable infectious diseases, more calls were able to be made compared to the k-mer approach as this approach used more sequence data (Figure 5). Similar to the K-mer based approach, *E. coli* was one of the most prevalent species identified. However, the same issue of misidentified *Burkholderia* sequences was observed.

### 3.5. Untargeted RNA sequencing on MinION using direct RNA sequencing Kit

The Direct RNA Sequencing Kit from Oxford Nanopore Technologies was selected for feasibility testing with wastewater-based epidemiology, as it sequences RNA molecules that have a polyA tail. The data gathered from the Illumina total RNA sequencing method demonstrates that even when samples have been enriched for viral RNA, most of the RNA is from bacteria in the samples. The bacterial sequences therefore drown out the viral signal, causing viral pathogens to not be detected. Therefore, a method that selects for polyA tails should further enrich for eukaryotic or viral sequences, as most bacterial RNAs do not contain polyA tails. Two locations were chosen for testing the utility of the MinION Direct RNA Sequencing method for wastewater biosurveillance: A and H. These locations were chosen because they represent two distinctive RNA sample types analyzed by the OCWN: A never produced a long amplicon peak in the Midnight RT PCR testing, although the RNA samples consistently had high RIN scores, while H consistently produced the long amplicon peak in the Midnight RT PCR testing, but never produced a RIN Score. Since RIN scores are based on the ribosomal RNA, this suggests that the H samples had been enriched for long viral RNA molecules that do not have rRNA, but A samples contained more eukaryotic RNAs and more fragmented RNA molecules. However, the direct RNA sequencing data shows very similar microbial profiles between H and A. While this method did reduce the complexity of the samples, contrary to the hypothesis that more viruses would be sequenced than bacteria, very few reads were identified as viral, and most of the classified sequences were determined to be human, bacteria, or fungi. Three options for What's In My Pot (WIMP) were used to analyze both samples (Table 5): Fastq WIMP, Fastq Antimicrobial Resistance WIMP, and Fastq WIMP (Human + Viral) with a 1% minimum abundance cutoff. The two viral reads identified in H were Alphabaculovirus and Chivirus. The seven viral reads identified in A were Mastadenovirus (2), Pandoravirus (2), Pahexavirus (1), Cyprinivirus (1), and Simplexvirus (1). Since the ONT direct RNA sequencing method did not enrich for more viral sequences, this method was not explored further.

**Table 5:** Distribution of read classification by WIMP option (Fastq, Antimicrobial Resistance (AMR), or human-viral) for H 12/26/21 and A 1/9/22 samples demonstrated that the direct RNA MinION sequencing method did not provide useful information on pathogens prevalence.

	**H 12/26/21**			**A 1/9/22**		
**Reads**	**Fastq**	**AMR**	**Human-viral**	**Fastq**	**AMR**	**Human-viral**
Analyzed	150,848	150,848	150,848	491,997	501,144	499,997
Classified	1,164	1,164	676	3,712	3,787	2,140
Unclassified	149,684	149,684	150,172	488,285	497,357	497,857
Human	654	654	673	1,991	2,033	2,130
Viral	–	–	2	–	–	7
Microbial	510	510	–	1,721	1,754	–

### 3.6. Illumina respiratory pathogen and AMR targeted sequencing panel

**Figure 6:**
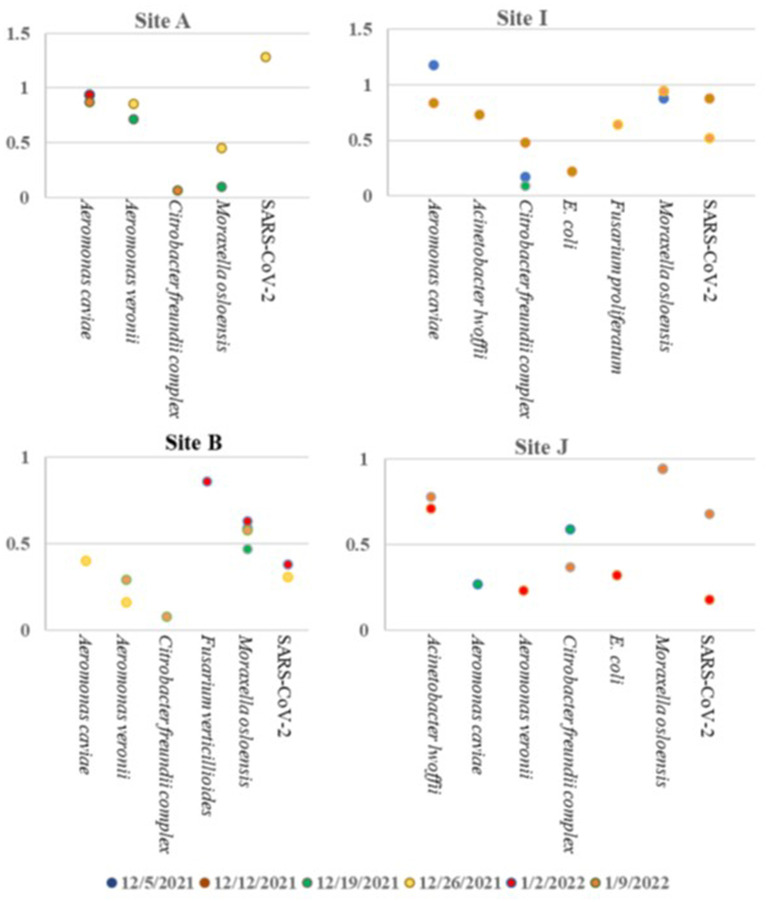
Pathogens identified in sites A, B, I, and J over time using the Illumina Respiratory Pathogen and AMR Targeted Sequencing Panel.

Based on the ambiguity of the untargeted sequencing data analysis, it was determined that a targeted approach may provide better confidence in a pathogen detection. Therefore, the Illumina Respiratory Pathogen and AMR (Antimicrobial Resistance) Targeted Sequencing Panel was tested for compatibility with wastewater samples for epidemiological purposes. This enrichment panel targets 282 respiratory pathogens, including 42 viruses (including SARS-CoV-2), 187 bacteria, and 53 fungi, and 1,218 AMR alleles covering resistance to Amoxicillin, Gentamycin, Amoxicillin-Clavulanate, Levofloxacin, Cefazolin, Meropenem, Cefepime, Oxacillin, Ceftriaxone, Sulfamethoxazole, Clindamyicn, Tetracycline, Colistin, Trimethoprim, Erythromycin, and Vancomycin. Twenty-eight wastewater RNA samples representing six sites (A, B, C, D, I, and J) were selected, prepared and sequenced alongside a negative control and a positive COVID RNA control on a single NextSeq Flow Cell. The RNA input for each sample was normalized to 100 ng starting material. As expected, SARS-CoV-2 was detected with high confidence, and no other pathogens or AMR genes were detected in the COVID Positive control, and no organisms or AMR genes were detected by the Explify software in the negative control. Of the 282 pathogens targeted by the panel, 54 pathogens were identified over the six sampling sites over time. The number of pathogens detected per week tended to be consistent within the same location: A had 2 ± 1, I had 4 ± 2, D had 30 ± 13, C had 22 ± 5, J had 4 ± 1, and B had 2 ± 2 pathogens detected each week. Interestingly, D (51 pathogens), and C (33 pathogens) had the most variety in pathogens identified (Table 6), while sites A, B, J, and I were less diverse (Figure 6). Since the D sewershed was the smallest population and C sewershed was the second largest population tested by this method, it demonstrates that the number of pathogens identified is not directly correlated with the population covered by sewershed sample. Since the input concentration was normalized, the total amount of RNA cannot explain difference in number of pathogens detected. More work with controlled inputs will need to be conducted to determine if the difference is caused by RNA extraction method, RNA quality, or methods used to enrich for viral vs. microbial RNA molecules. The most commonly detected organism in all sites was *Moraxella osloensis*, being found in 27 of the 28 samples tested.

**Table 6:** Pathogens detected by the illumina respiratory pathogen and AMR panel in sites D and C over time.

**Pathogens**	**D**				**C**					**COVID +control**
	**12/5**	**12/12**	**12/19**	**1/9**	**12/5**	**12/12**	**12/19**	**1/2**	**1/9**	
*Acinetobacter baumannii*		1.28	0.41			1.2				
*Acinetobacter lwoffii*	1.28	1.54	1.54	1.54	1.54	1.54	1.53	1.54	1.54	
*Acinetobacter pittii*		0.14				0.58				
*Actinomyces graevenitzii*		0.44				1.33				
*Aeromonas caviae*	1.99	2.00	2.00	2.00	2.00	2.00	1.99	1.99	2.00	
*Aeromonas hydrophila*	0.51	1.18	0.58	0.39	0.56	1.17	0.94	1.17	0.94	
*Aeromonas sobria*		0.17								
*Aeromonas veronii*	1.30	1.51	1.50	1.51	1.51	1.51	1.18	1.24	1.50	
*Aspergillus versicolor*	0.87									
*Bacteroides fragilis*	2.34	2.34	2.34	2.34	2.34	2.34	2.34	2.34	2.34	
*Burkholderia cepacia*			0.29		0.30	2.51	2.10	2.50	2.51	
*Citrobacter freundii*	2.10	2.02	2.02	2.15						
*Cronobacter sakazakii*		1.91	0.80							
*Delftia acidovorans*		0.79	0.65							
*Enterobacter cloacae*	1.76	1.76	1.76	1.65	1.76	1.76	1.75	1.70	1.76	
*Enterococcus faecalis*		2.33								
*Enterococcus faecium*		2.22	0.53			0.51				
*Escherichia coli*	1.42	1.42	1.42	1.44	1.42	1.42	1.69	1.45	1.42	
*Finegoldia magna*		0.18								
*Gordonia bronchialis*	0.49	1.30	1.30	1.30		0.13				
*Haemophilus parainfluenzae*		0.18								
*Hafnia alvei*		1.05								
*Klebsiella aerogenes*		2.58								
*Klebsiella oxytoca*	0.94	1.57	1.59	1.46	1.56	1.54	1.57	0.97	1.58	
*Klebsiella pneumoniae*	1.58	1.57	1.56	1.58	1.56	1.57	1.57	1.54	1.61	
*Klebsiella quasipneumoniae*		2.04	2.04	1.45	2.05	2.05	1.97	1.55	2.06	
*Klebsiella variicola*		1.97	1.96	1.41	1.96	1.96	1.77	1.95	1.36	
*Leclercia adecarboxylata*		0.05	0.95			0.47				
*Moraxella osloensis*	0.94	0.94	0.94	0.94	0.94	0.94	1.54	0.94	0.94	
*Morganella morganii*		1.94	0.72			0.56				
*Mycobacterium avium*	1.49	2.15				1.22	2.25	0.11		
*Mycobacterium fortuitum*		1.81	1.81	1.82						
*Mycobacterium gordonae*		1.27	0.28							
*Mycobacterium simiae*		2.07		0.88						
*Mycobacteroides chelonae*		0.30	0.21			0.35				
*Ochrobactrum anthropi*		1.58	1.23			0.20				
*Pantoea agglomerans*						0.75				
*Pediococcus acidilactici*		2.14			0.66					
*Prevotella buccae*		0.07								
*Pseudomonas aeruginosa*		1.17								
*Pseudomonas fluorescens*		0.16		0.12						
*Pseudomonas stutzeri*		1.37	0.99		0.40	1.30	1.29	1.33	1.34	
*Raoultella ornithinolytica*	1.81	1.67	1.81	1.81	1.81	1.81	1.81	1.81	1.81	
*Raoultella planticola*	1.19	1.64	1.64	1.02	1.60	1.64	1.61	1.62	1.64	
*Rothia mucilaginosa*		0.27	0.73							
SARS-CoV-2	1.15									2.17
*Serratia marcescens*	0.21	1.50	1.41	1.42		1.22				
*Shewanella putrefaciens*	0.55	1.20					1.49		1.24	
*Stenotrophomonas maltophilia*	0.10	1.60	1.30	0.61	0.50	1.32	0.59	0.58	0.73	
*Streptococcus anginosus*		1.62								
*Veillonella parvula*		1.23								
*Yersinia enterocolitica*		1.97	1.77			1.18			1.81	

red text =Antibiotic Resistance. Gray is undetected, Red = High, Orange = Medium, Yellow = Low detection.

Using this panel, the *Burkholderia* issue observed in untargeted sequencing was resolved: only *Burkholderia cepacia* complex was identified, with a confidence score which removes the ambiguity. Furthermore, the targeted approach enabled detection of viral pathogens present in the wastewater, such as SARS-CoV-2 as detected in some of the samples. However, this data set demonstrates the need to determine the limit of detection for each pathogen in the panel to better understand the parameters necessary to enable wastewater pathogen surveillance with high confidence in what is and is not detected in each sample.

The information provided by the panel on antimicrobial resistance markers is also very interesting to public health. Out of the 22 drug classes covered, antimicrobial resistance markers were found against 13: Aminoglycosides, Carbapenems, Beta-Lactam + Beta-Lactamase Inhibitors, Cephalosporins, Diaminopyrimidine, Fluoroquinolones, Glycopeptides, Lincosamides, Macrolides, Oxazolidinones, Penicillins, Sulfonamides, and Tetracyclines. Six different organisms identified had associated antibiotic resistances identified (*Stentrophomonas maltophilia, Klebsiella pneumoniae, E. coli, Enterobacter cloacae complex, Acinetobacter baumannii*, and *Pseudomonas aeruginosa*), and several other AMR genes were detected that were not associated with pathogens detected by the panel. The relative abundance expressed as Reads per kilobase of transcript per Million reads mapped (RPKM) is provided for each AMR gene identified by week at each site is presented in Supplementary Table 1. Five AMR genes were consistently found in all samples tested: AAC(6')-Ib-cr (aminoglycoside), GES-5 (Carbapenem/Beta-Lactam +/Beta-Lactamase Inhibitor/Cephalosporin/Penicillin), *mphE* and *msrE* (Macrolides) and *sul1* (sulfonamides). In 17 of the 28 wastewater samples tested by targeted hybridization, either *mphE* and *msrE* were the most abundant AMR gene detected. For the other 11 samples, 10 had aminoglycoside resistance gene marker *RRS (1402C*>*A)* and one sample had *GES-5* as the most abundant AMR gene detected.

## 4. Discussion

Wastewater based surveillance of human pathogens would enable monitoring and early detection of epidemics before they reach pandemic levels, enabling quick response to inhibit the spread of deadly bacteria or viruses. However, the traditional approaches to metagenomic analysis, such as shotgun metagenomics or 16S amplicon sequencing do not interrogate the RNA of the sample and thereby miss any RNA viruses such as SARS-CoV-2 that could be present in the sample because these pathogens do not have a 16S gene or a DNA based genome. Methods exist for metatranscriptomic analysis, which look at untargeted sequencing of all RNA present in a sample. Metatranscriptomics has another advantage of metagenomic and 16S amplicon sequencing based methods, as RNA is produced from active or viable microbes, and demonstrates what genes are being expressed. Therefore, any AMR gene detected from RNA based methods are actively being expressed in the microbial community. While metatranscriptomics is able to detect eukaryotic, bacterial, and viral pathogens, several studies have shown that the untargeted methods available are not adequate to detect unknown pathogens with small genomes such as viruses on a large scale at a reasonable cost (20, 24, 32, 33). Surveillance of viruses in wastewater has been mainly conducted using targeted approaches such as amplicon sequencing for particular pathogen genomes such as SARS-CoV-2 or norovirus (4, 5, 17, 20, 24, 34) or through use of viral concentration methods which remove all bacteria and eukaryotic cells prior to sequencing, thereby removing information on other potential pathogens (3, 35). Furthermore, wastewater is comprised of intact as well as cellular debris, and therefore, filtration and concentration methods do not enrich enough for viruses to enable consistent detection by non-targeted sequencing methods (20, 33). The data presented in this report demonstrates that wastewater can be used to track several pathogens simultaneously over time across the state of Ohio using RNA extracts already gathered through the OCWMN. However, several gaps were identified in the wastewater sequencing based epidemiology methodology that need to be addressed prior to implementation on a broad scale, congruent with other studies (20, 33). To be cost efficient and enable high throughput wastewater based biosurveillance, a single method must be developed that can detect both bacterial and viral pathogens without compromising detection of any pathogenic group. Here we show that not all sequencing methods were equally able to detect pathogens of interest, and the RNA extraction method used influences the data output from sequencing. Targeted hybridization was able to detect bacterial, fungal, and viral pathogens in the wastewater, and therefore is a viable singular method for known pathogen surveillance. Still, the methods used to conduct total pathogen biosurveillance need to be optimized and standardized across each network to enable surveillance of all reportable diseases. First, positive controls for each pathogen targeted must be developed to enable a baseline for comparison for each target. Second, a targeted hybridization panel needs to be identified or expanded to include all reportable infectious diseases and those of interest to the CDC to ensure detection of known viral, bacterial, and eukaryotic pathogens. The panel will then need to be tested on RNA extracts from the positive control spiked into wastewater from different sources with different RNA extraction methods to determine the optimal workflow(s) to detect all pathogens. Finally, the limit of detection for each pathogen in wastewater will need to be determined prior to implementation.

Of the three methods for detection of reportable pathogens, the MinION direct RNA sequencing method performed the worst, not detecting a single pathogen. While Illumina untargeted RNA sequencing was able to detect pathogens of interest, due to the random sequencing of potentially shared genomic regions between pathogens and non-pathogen near neighbors, confidence in pathogen/non-pathogen calls was not high enough to enable implementation on a broad scale. Another study effectively used the MinION to quantify and assess the removal of bacterial pathogens and antibiotic resistance genes (ARGs) in wastewater treatment plants using metagenomic sequencing (32). However, the method employed in that study would not detect viruses that have human health consequences such as SARS-CoV-2 or influenza A. Targeted hybridization, however, was able to provide direct detection of pathogens and antimicrobial resistance genes with high confidence in the calls. Macrolide resistance genes *mphE* and *msrE* were found in all 28 samples tested and were the most abundant AMR genes detected in 17 samples tested. This finding is consistent with a recent report which analyzed AMR genes in a river downstream from a wastewater treatment plant in Ohio, where *mphE* and *msrE* were the most abundant AMR genes identified in the river water (36). The easy-to-use Explify bioinformatics tool, also makes it more amenable to users who may not be trained in the use of command line bioinformatics tools that are generally used for untargeted sequencing analysis.

Targeted hybridization was found to be more useful than untargeted RNA sequencing because the amount of nucleic acid from non-pathogenic microbes and humans in the sample obscured the pathogen signal, causing pathogen signal to often be below the limit of detection. Despite efforts to enrich by subtractive hybridization or through viral enrichment conducted by the RNA extraction laboratories, most sequences were human, non-pathogenic bacteria or bacteriophage. Furthermore, relatedness between non-pathogens and pathogens can confound taxonomic classification in short-read untargeted RNA sequencing, making it difficult to determine if a pathogen is present or absent. Untargeted methods align or map sequences 100–150 bp in length to a reference database. Given that an average bacterial genome is ~5 million bp, and bacteria within the same genera can have up to 50% genomic similarity, it is often extremely difficult to distinguish between species with this method. Since untargeted metatranscriptomic sequencing does not capture full genomes, and instead only sequences random fragments from each genome present, many sequences align to regions in common between closely related pathogens and non-pathogens. This causes ambiguity in determination of pathogen presence or absence as observed in *E. coli* and *Burkholderia*. Targeted methods, on the other hand, can be designed to target regions specific to the pathogenic strains or species, enabling detection and the ability to discern between such closely related species, as demonstrated by the Illumina Respiratory Pathogen and AMR Targeted Sequencing Panel sequencing results.

The use of the MinION for long read SARS-CoV-2 genomics was also assessed in this work. It was found that although consensus calls could be made on the major variants present in the samples, that the RNA extraction method drastically affects the ability of sequencing of long reads. Only four locations tested: E, F, G, and H consistently produced sequencing results, all of which were extracted using method 4. Therefore, if MinION sequencing were to replace Illumina sequencing of SARS-CoV-2, the RNA extraction method will need to be standardized to method 4 to enable long read sequencing from all sites. Utilization of the MinION could enable faster, decentralized analysis of samples in real time at wastewater treatment facilities. The device is smaller than a cell phone and runs off a laptop computer. Therefore, if the RNA extraction method is optimized to produce consistent data on the MinION for SARS-CoV-2 variant calling, the time between sampling and answer could be reduced by 3 days, since the library preparation is much faster than that used for Illumina sequencing, and variant calls are automatically generated within an hour of initiating sequencing, therefore, a trained bioinformatician is not needed for analysis, unlike with Illumina sequence data. One caveat of MinION sequence analysis is that the variant call will be made from a consensus of the data and will not identify multiple variants that may be present in the population-based wastewater data, without human intervention.

To enable established COVID wastewater tracking networks to transition into a pathogen surveillance network, we recommend development of a targeted hybridization panel to detect all reportable disease pathogens and AMR genes from RNA extracts. Future work will include development and validation of a targeted hybridization panel to detect all reportable disease pathogens of interest, development of control materials, and optimization of RNA extraction methods to ensure pathogen detection from different wastewater samples with known limit of detection, sensitivity, and specificity for each pathogen.

## Data availability statement

The datasets presented in this study can be found in online repositories. The names of the repository/repositories and accession number(s) can be found at: https://www.ncbi.nlm.nih.gov/genbank/, PRJNA924011.

## Author contributions

RS planned and supervised the study, helped with laboratory preparations, analysis, and wrote the manuscript. AM-S and CM conducted the bioinformatic analysis of the data and contributed to the writing and figure generation for the manuscript, with CM contributing k-mer analysis, and AM-S conducting contig based approach for untargeted metatranscriptomics, analysis of MinION sequencing data, and the analysis of the targeted hybridization data. LC conducted the laboratory preparation of samples for Illumina and MinION based sequencing and contributed to the writing of the manuscript. AS assisted with laboratory preparation of samples. All authors contributed to the article and approved the submitted version.
